# Field Evaluation of a High Throughput Loop Mediated Isothermal Amplification Test for the Detection of Asymptomatic *Plasmodium* Infections in Zanzibar

**DOI:** 10.1371/journal.pone.0169037

**Published:** 2017-01-17

**Authors:** Berit Aydin-Schmidt, Ulrika Morris, Xavier C. Ding, Irina Jovel, Mwinyi I. Msellem, Daniel Bergman, Atiqul Islam, Abdullah S. Ali, Spencer Polley, Iveth J. Gonzalez, Andreas Mårtensson, Anders Björkman

**Affiliations:** 1 Centre for Malaria Research, Department of Microbiology, Tumor and Cell Biology, Karolinska Institutet, Stockholm, Sweden; 2 Unit of Infectious Diseases, Karolinska University Hospital, Stockholm, Sweden; 3 Foundation for Innovative New Diagnostics (FIND), Geneva, Switzerland; 4 Zanzibar Malaria Elimination Programme, Ministry of Health, Zanzibar, Tanzania; 5 Department of Clinical Parasitology, Hospital for Tropical Diseases, University College London Hospitals, NHS Foundation Trust, London, United Kingdom; 6 Department of Women’s and Children’s Health, International Maternal and Child Health (IMCH), Uppsala University, Uppsala, Sweden; Université Pierre et Marie Curie, FRANCE

## Abstract

**Background:**

New field applicable diagnostic tools are needed for highly sensitive detection of residual malaria infections in pre-elimination settings. Field performance of a high throughput DNA extraction system for loop mediated isothermal amplification (HTP-LAMP) was therefore evaluated for detecting malaria parasites among asymptomatic individuals in Zanzibar.

**Methods:**

HTP-LAMP performance was evaluated against real-time PCR on 3008 paired blood samples collected on filter papers in a community-based survey in 2015.

**Results:**

The PCR and HTP-LAMP determined malaria prevalences were 1.6% (95%CI 1.3–2.4) and 0.7% (95%CI 0.4–1.1), respectively. The sensitivity of HTP-LAMP compared to PCR was 40.8% (CI95% 27.0–55.8) and the specificity was 99.9% (CI95% 99.8–100). For the PCR positive samples, there was no statistically significant difference between the geometric mean parasite densities among the HTP-LAMP positive (2.5 p/μL, range 0.2–770) and HTP-LAMP negative (1.4 p/μL, range 0.1–7) samples (p = 0.088). Two lab technicians analysed up to 282 samples per day and the HTP-LAMP method was experienced as user friendly.

**Conclusions:**

Although field applicable, this high throughput format of LAMP as used here was not sensitive enough to be recommended for detection of asymptomatic low-density infections in areas like Zanzibar, approaching malaria elimination.

## Background

Ultra-sensitive, field-friendly high-throughput diagnostic tools are needed for accurate detection of asymptomatic parasite carriers in malaria pre-elimination settings. These often low parasite density infections constitute a large proportion of the parasite reservoirs in low-endemic settings [1–3] with parasitemias often falling below the detection limit of both microscopy and malaria rapid diagnostic tests (mRDT)[4]. Thus, more sensitive methods, i.e. nucleic acid based techniques such as PCR or loop-mediated isothermal amplification (LAMP) are needed [5].

LAMP has been shown in previous field studies to be a sensitive, robust and fast method, requiring less sophisticated equipment than PCR [6–8]. LAMP is also among the techniques recommended for malaria diagnosis in low transmission settings by the WHO Global Malaria Programme [9]. The Loopamp^TM^ MALARIA Pan/Pf detection kit (Eiken Chemical Company, Japan) has recently been evaluated in two studies in Zanzibar, a malaria pre-elimination area [10,11], following a simple but relatively time consuming boil and spin DNA extraction method. Hence, more efficient, field friendly high throughput (HTP) techniques for DNA extraction are needed if LAMP is going to be a useful tool in for example mass screen and treat programmes [11].

The department of Clinical Parasitology, Hospital of Tropical Diseases in London UK, together with 42T, a medical device prototyping company and Porvair, a company specialized in filtration, have developed a HTP system for DNA extraction compatible with subsequent LAMP reaction. The HTP-LAMP system is a specially designed series of consumables and hardware for processing the extraction from up to 96 samples in parallel. The final eluate containing *Plasmodium* DNA can be used directly for the malaria LAMP reaction and results can be obtained in two hours. The performance of the HTP-LAMP assay has been evaluated using clinical samples in a laboratory setting, as shown in accompanying publication by Perera et al [12].

We herein report an evaluation of the field performance of the HTP-LAMP system for DNA extraction and detection of malaria infections among asymptomatic individuals in Zanzibar, including an assessment of its user-friendliness and time-to-result when performed by local laboratory technicians.

## Materials and Methods

### Study site

The study was conducted in two districts of Zanzibar, North A (Unguja Island) and Micheweni (Pemba Island). Community based cross-sectional surveys have been regularly performed since 2003 in the two areas to assess the impact and uptake of wide scale deployment of combined malaria control interventions. In 2013 the malaria prevalence was estimated to be 0.4% by RDT and 2.3% by PCR in the study area [13].

### Study design, conduct and treatment

The field evaluation of HTP-LAMP was performed as an integrated part of a community based cross-sectional survey conducted during the mid-rainy season, i.e. the first two weeks of June, 2015. Eight teams, four per district with two enumerators each, visited randomly pre-selected households during 12 days. All members in selected households ≥6 months of age, with no signs of severe disease, who provided informed/proxy consent were eligible. All study participants underwent a finger prick blood sampling for mRDT (First Response® Malaria Ag Combo HRP2/pLDH (Premier Medical Corporation Limited, India) and HTP-LAMP test and PCR tests. The mRDT results together with demographic data were instantly entered on Nexus 7 tablets. All HTP-LAMP analyses were performed the day after sampling at Kivunge Hospital, North A district. Artemisinin combination therapy was provided to mRDT positive individuals the same day and to LAMP positive individuals within 72 hours of sample collection.

### Sample collection and storage for HTP-LAMP and PCR

In addition to blood sampling for the mRDT, each study participant provided two blood spots, approximately 30 μL each on filter papers (Whatman 3MM) and 2x 20 μL of blood on specifically designed filter devices [14]. The filter papers and filter devices were pre-labelled with matching barcodes and placed in structured collection boxes to air dry. Samples collected in North A district were transported to Kivunge at the end of each day. Samples collected in Micheweni district were transported by air to Unguja Island the following morning and arrived at Kivunge hospital around noon.

The dried blood spots (DBS) collected on filter papers were packed individually each morning in zipped plastic bags containing a desiccants pouch and later sent to Karolinska Institutet, Sweden, for PCRs and repeat LAMP analyses.

### Training of study staff

All field coordinators and enumerators received a five-day pre-study training covering data collection, blood sampling and mRDT performance. Two lab technicians, without previous experience in molecular laboratory work, underwent a structured HTP-LAMP training with one day of theory and three days of hands-on practical sessions.

### Ethical considerations

Written informed consents/proxy consents by parents/ guardians for children were obtained from all participants prior to study enrolment. Ethical approval was granted by the Zanzibar Medical Research and Ethics Committee (ZAMEC/0002/APRIL/005) and the Regional Ethics Committee, Stockholm, Sweden (2013/836-32).

### HTP-extraction and LAMP amplification

All equipment and consumables for HTP-extraction, including heating block for blood lysis, heat incubator, shaker and vacuum manifold for sample transfer packed in a specially designed transport case, as well as PURE kits for the HTP-DNA extraction were sent to Zanzibar at ambient temperature and set up at a basic laboratory facility in Kivunge hospital. The consumable kits for each set of 96 samples included three plates, one each for lysis, purification and elution as well as reagents and sample recording chart. The HTP-extraction method was conducted according to manufacturer’s guidelines and as described in the accompanying article [12]. In brief, one collection device containing 20 μL dried capillary blood was cut into each of the 96 deep wells of the lysis plate containing 500 μL lysis buffer, leaving two wells empty for the LAMP controls. Each well was plugged with a foam plug. The lysis plate was left on a shaker for 10 min at 1000 revs per minute (rpm), followed by incubation at 95°C for 20 min. The lysis plate was then placed on top of the purification plate in the vacuum manifold, for transferring of the lysate under -20 mm/Hg pressure. The purification plate containing a white powder and the lysate was left on the shaker for 10 min at 1000 rpm, and then placed on top of the elution plate in the vacuum manifold for the final transfer. The purified DNA in the elution plate was immediately used for the LAMP amplification.

**Fig 1:**
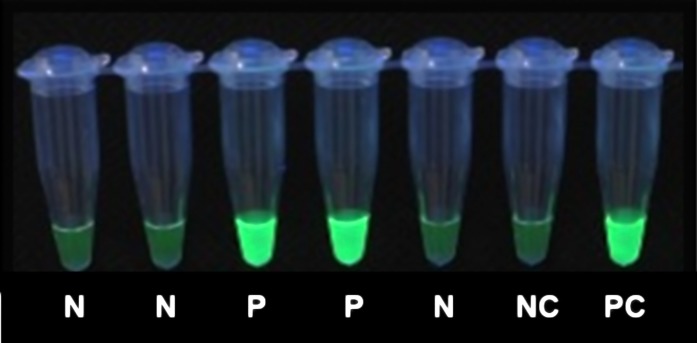
Detection of LAMP result under UV light. N- negative, P- positive, NC-negative control, PC- positive control.

The LAMP assay was performed using the Loopamp^TM^ MALARIA *pan*-Detection kit as described previously [10,11,15] with minor modifications. The 30μL eluate from the extraction plate was transferred directly into the LAMP reaction tubes using an 8-channel pipette. A negative and a postive control were included in every 96 samples. Two heatblocks at 65°C were used for the LAMP assay (to fit the 12 strips of eight reaction tubes); the amplification was terminated by tranferring the samples to heatblocks set at 80°C for five minutes. Results were read simultaneously by two persons using a UV lamp (Fig 1), and in case of disagreement a third person´s reading was decisive. Samples positive for *Pan-*LAMP (all *Plasmodium* species) were retested using the *Pf (P*. *falciparum)*-specific LAMP kit. The detection limit for the *Pan/Pf* Detection kit has been determined to be 2–5 parasite per microliter (p/μL) for both *Pan* and *P*. *falciparum* [7,16,17]. The HTP-LAMP has shown a detection limit of 1 p/μL when a turbidimeter was used for detection [12].

### Evaluation of HTP-LAMP performance

The PCR and repeat LAMP analyses used for evaluation of HTP-LAMP were performed at Karolinska Institutet, Sweden, within six months after the HTP-LAMP assays. The study researchers performing the initial PCR screening were blinded to the HTP-LAMP results.

#### DNA extraction for PCR analysis

DNA was extracted from two Ø 3 mm punches (≈ 6–10 μL blood) of the DBS on filter papers using the Chelex^®^-100 extraction method [18,19] with some modifications. In brief, two punches were incubated in 0.5% Saponin in PBS for five min. and washed in PBS for 30 min.at room temperature. The samples were then incubated for ten minutes at 95°C in 100 μL of 10% Chelex-100 (Bio-Rad Laboratory, USA). After centrifugation, 45 μL of the DNA eluate was transferred into 96-well plates and stored at -20°C until use.

#### PCR and LAMP analysis

All Chelex-extracted samples were screened for parasite DNA with a single round of SYBR Green real-time PCR (cytb-qPCR) [19]. The PCR was conducted in 384 well plates with 2 μL DNA template in a total volume of 15 μL. Positivity was confirmed by gel-electrophoresis on a 1.5% agarose gel stained with GelRed (Biotium Inc., USA). Positive and negative controls were included in each run. The cytb-qPCR was repeated for positive samples to determine *Plasmodium* species using a restriction fragment length polymorphism (RFLP) assay [19]. Results were documented with a GelDoc™ system (Bio-Rad Laboratory, USA). The PCR detection limit for the cytb-qPCR assay has previously been determined to 1 p/μL for *P*. *falciparum* [19].

In total 66 samples positive in either HTP-LAMP (n = 22) or initial cytb-qPCR screening (n = 44), were re-extracted by the Chelex method in duplicate. The cytb-qPCR was repeated in duplicate for each re-extraction (i.e. four repeat PCRs). A PCR result was considered finally positive if confirmed positive in ≥2 PCRs including cytb-qPCR, species determination and parasite density estimates. These confirmed PCR positive results were used as gold standard for performance evaluation of HTP-LAMP.

To evaluate the effect of DNA extraction method on the LAMP outcome, all 66 Chelex re-extracted samples were also analysed with the same *Pan-*LAMP kit to assess the agreement between HTP- and Chelex-extracted (Ch-) LAMP results.

#### Parasite density estimation by qPCR

A modified version of a probe-based qPCR method targeting *Plasmodium* 18s rRNA against standards of known parasite densities [16,20] was used to estimate parasite density of PCR positive samples. Parasite density of cultured *P*. *falciparum* laboratory strain 3D7 was estimated by microscopy. Packed cells from the culture was diluted in human serum to achieve a haematocrit of ~50% and then diluted with uninfected whole blood to densities of 20,000 to 0.02 p/μL and spotted on filter papers. Parasite densities of samples were determined against standards included in each PCR run. The parasite density of each sample was estimated from the mean of three qPCR repeats. The standard dilutions were evaluated against the WHO International Standard for *P*. *falciparum* Nucleic acid Amplification Techniques [21].

#### Estimation of blood sample input for respective molecular method

HTP extraction for LAMP: 20 μL blood was lysed and eluted in 500 μL lysis buffer. 30 μL of the eluate was added to LAMP reaction tube, corresponding to approximately 1 μL blood.

Chelex extraction for LAMP: 6–10 μL blood (from two 3mm punches) was extracted in 100 μL Chelex. 5 μL of the eluate was added to 25 μl of water (1:6 dilution) and the full volume was added to reaction tube, corresponding to approximately 1 μL blood.

Chelex extraction for PCR: 6–10 μL blood (from two 3mm punches) was extracted in 100 μL Chelex. 2 μL of the eluate was added to the PCR reaction, corresponding to approximately 0.2 μL blood.

#### Assessment of field performance of the HTP-LAMP method

One person from the research team supervised the work of the two technicians during the survey. Eventual problems, number of samples analysed and time to result was reported. At the end of the survey the technicians were asked regarding their experiences of using the HTP-LAMP method.

### Statistical analysis

Statistical analysis was conducted using STATA 12 (Stata Corp, Texas, USA). The diagnostic performance of HTP-LAMP (index test) was assessed using confirmed PCR as gold standard and included sensitivity, specificity and predictive values with 95% confidence intervals (CI). Pairwise determination for non-equivalence between the methods was assessed by the McNemar´s test. Parasite densities in HTP-LAMP positive and negative samples were compared by two-sample Wilcoxon rank-sum (Mann-Whitney) test. Kappa agreement between HTP-LAMP and Ch-LAMP methods was performed.

The study was performed in compliance with the updated version of Standards for Reporting of Diagnostic Accuracy (STARD) [22].

## Results

### Study population

Some 3021 individuals in 685 households consented to participate in the study. The median age of the participants was 14 years (range 0–90 years), of whom 1782 (59%) were females. RDT, HTP-LAMP and PCR results were available from 3021, 3019 and 3009 individuals, respectively.

### mRDT results

A total of 13/3021 (0.4%; 95%CI 0.2–0.8) individuals were positive for *Pf*-HRP2, none were pLDH positive. Among the 13 RDT positives, 10 (77%) were also positive by PCR and 8 (62%) by HTP-LAMP.

### HTP-LAMP results

**Fig 2:**
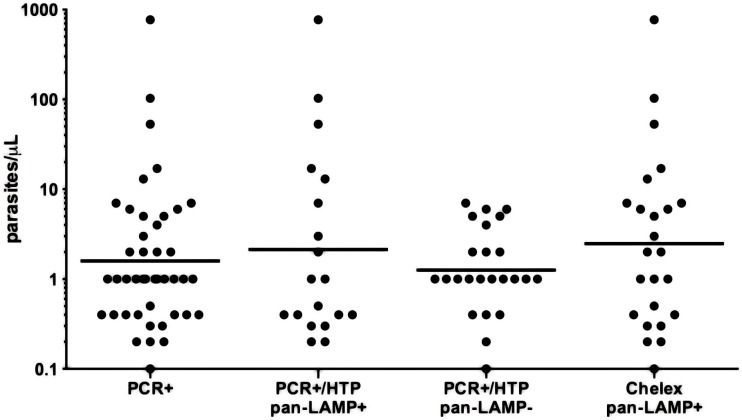
Distribution of quantitative PCR determined parasite densities. pan- *Pan plasmodium*, Geometric mean values are indicated by horizontal lines. Geometric mean values are for PCR + 1.8 p/μL (range 0.1–770), for PCR+/ pan HTP-LAMP + 2.5 p/μL (range 0.2–770), for PCR+/ pan-HTP-LAMP– 1.4 p/μL (range 0.1–7) and for Chelex pan-LAMP + 3.4 p/μL (range 0.1–770).

A total of 22/3019 (0.7%; 95%CI 0.4–1.1) samples were positive by *Pan*-LAMP of which 16 (73%) were also positive for *Pf*-LAMP (Table 1). Three of the six *Pan*-LAMP positive/*P f*-LAMP negative were confirmed as *P*. *malariae* by PCR. The qPCR determined geometric mean parasite density for *Pan*-LAMP was 2.5 p/μL (range 0.2–770) (Fig 2). For *Pf*-LAMP the geometric mean was 3.5 p/μL (range 0.2–770).

**Table 1:** Characterization of HTP-LAMP positive blood samples (N = 22).

HTP-LAMP (pan)	HTP-LAMP (P.f)	PCR^1^	Chelex LAMP	Species^2^	Parasite density ^3^ (p/μL)
**+**	+	+	+	P. f	770
**+**	+	+	+	P. f	103
**+**	+	+	+	P. f	53
**+**	+	+	+	P. f	17
**+**	+	+	+	P. f	13
**+**	+	+	+	P. f	7
**+**	+	+	+	P. f	3
**+**	+	+	+	P. f	1
**+**	+	+	+	P. f	0.5
**+**	+	+	+	P. f	0.4
**+**	+	+	+	P. f	0.4
**+**	+	+	-	P. f	0.4
**+**	-	+	+	P. f	0.3
**+**	+	+	-	P. f	< LD
**+**	+	+	-	P. f/P. m	0.4
**+**	-	+	+	P. f/P. m	0.3
**+**	-	+	+	P. m	2
**+++**	-	+	+	P. m	1
**+**	-	+	+	P. m	0.2
**+**	+	+	-	IR	0.2
**+**	+	-	-	ND	< LD
**+**	-	-	-	ND	< LD

Pan-*Pan plasmodium*, P.f- *P*.*falciparum*, P.m- *P*. *malariae*

p/μL- parasites /microliter, IR-inconclusive result, ND- not determined, <LD–below limit of detection

^1^Cytochrome b real time PCR

^2^Cytochrome b real time PCR-RFLP assay

^3^ by quantitative PCR.

### PCR results

A total of 54/3009 samples were positive in the initial screening by cytb-qPCR and 39/54 (72%) were confirmed positive by either species identification, quantification or re-extraction and repeat PCR assays. In addition, 10/12 HTP-LAMP positive and PCR screening negative samples were confirmed PCR positive after re-extraction and repeat cytb-qPCR assays. Thus, in total 49 (1.6%; 95%CI 1.3–2.4) samples were finally considered PCR positive. Three samples had inconclusive species results, and of the remaining 46 samples, 39 (85%) were *P*. *falciparum* mono-infections, five (11%) *P*. *malariae* mono-infections and two (4%) *P*. *falciparum/ P*. *malariae* mixed infections. The qPCR determined geometric mean parasite density was 1.8 p/μL(range 0.1-770/μL) (Fig 2). All five *P*. *malariae* mono infections had densities of ≤ 2p/μL. Parasite densities could not be determined in five samples due to negative q-PCR (below detection limit) (Table 2).

**Table 2:** Characterization of HTP-LAMP versus PCR discordant samples for diagnosis of *Plasmodium* infection (N = 31).

HTP-LAMP (pan)	PCR^1^	Chelex LAMP	Specie^2^	p/μL^3^
-	+	+	P.f	7
-	+	+	P.f	6
-	+	+	P.f	6
-	+	-	P.f	5
-	+	+	P.f	5
-	+	-	P.f	4
-	+	-	P.f	2
-	+	-	P.f	2
-	+	+	P.f	2
-	+	+	P.f	1
-	+	-	P.f	1
-	+	-	P.f	1
-	+	-	P.f	1
-	+	-	P.f	1
-	+	-	P.f	1
-	+	-	P.f	1
-	+	-	P.f	1
-	+	-	P.f	1
-	+	-	P.f	1
-	+	-	P.f	0.4
-	+	-	P.f	0.4
-	+	-	P.f	0.4
-	+	+	P.f	0.2
-	+	-	P.f	<LD
-	+	-	P.f	<LD
-	+	-	P.f	<LD
-	+	+	P.m	0.1
-	+	-	P.m	<LD
-	+	-	IR	<LD
+	**-**	**-**	ND	ND
+	**-**	**-**	ND	ND

pan-*Pan plasmodium*, P.f- *P*.*falciparum*, P.m- *P*. *malariae*

p/μL- parasites/microliter, IR-inconclusive result, ND- not determined, <LD–below limit of detection.

^1^ Cytochrome b real time PCR.

^2^ Cytochrome b real time PCR-RFLP assay.

^3^ by quantitative PCR.

### Ch-LAMP results

The *Pan*-LAMP kit was repeated on the 66 Chelex re-extracted samples that had also been repeated with PCR. Out of these, 24 were positive by Ch-LAMP. All Ch-LAMP positive samples were confirmed PCR positive. The sensitivity of Ch-LAMP versus PCR was 49% (24/49, 95% CI 34.4–63.7). The geometric mean parasite density of Ch-lamp was 3.4 p/μL (Fig 2).

### HTP-LAMP performance

**Fig 3:**
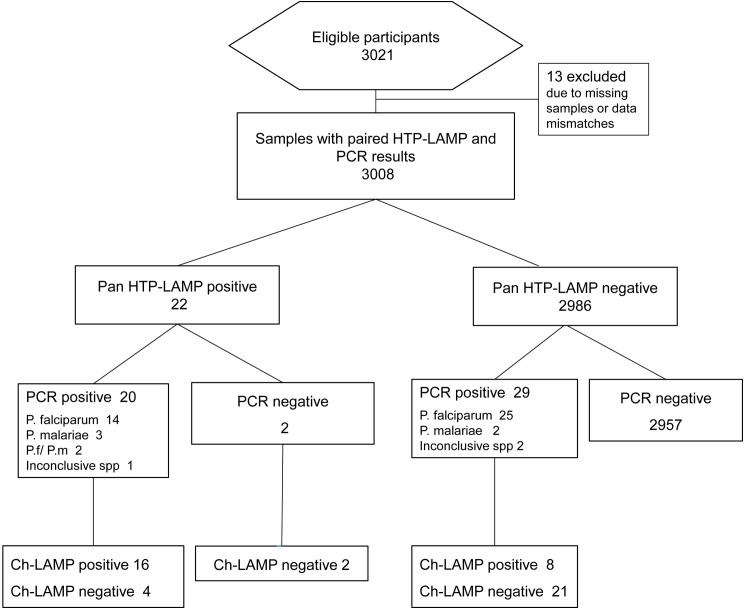
Flow chart of high-throughput (HTP)-LAMP evaluation. Results of Pan *Plasmodium* HTP-LAMP, confirmed PCR and Chelex LAMP (Ch-LAMP) are summarized. spp-species.

A total of 3008 samples had paired HTP-LAMP and PCR data and thus used for the evaluation of HTP-LAMP diagnostic performance (Fig 3). Concordant positive results by PCR and HTP-LAMP were found in 20 samples and concordant negative results in 2957 samples. Discordant results were found in 31 samples, 29 were confirmed positive by PCR but negative by HTP-LAMP and two samples identified as positive by HTP-LAMP were negative by PCR (Fig 3 and Table 2). One of these two samples was also positive by *Pf-*LAMP (Table 1). The sensitivity, specificity, positive and negative predictive values were 40.8% (95% CI 27.0–55.8), 99.9% (95% CI 99.8–100), 90.9% (95% CI 70.8–98.9) and 99.0% (95% CI 98.6–99.3), respectively. Comparison with McNemar´s test showed a significant difference between the two methods (*p*< 0.001) (Table 3).

**Table 3:** Diagnostic accuracy of Malaria pan HTP-LAMP compared to PCR for 3008 field samples.

	PCR +	PCR -	Total
**HTP-LAMP +**	20	2	22
**HTP-LAMP -**	29	2957	2986
**Total**	49	2959	3008
**p** < 0.001*			
**Sensitivity**	40.8% (95%CI 27.0–55.8%)		
**Specificity**	99.9% (95%CI 99.8–100%)		
**Positive predictive value**	90.9% (95%CI 70.8–98.9%)		
**Negative predictive value**	99.0% (95%CI 98.6–99.3%)		

* by McNemar´s test.

Among the PCR positive/HTP-LAMP negative samples the geometric mean parasite density was 1.4 p/μL (range 0.1–7) and among PCR positive/HTP-LAMP positives 2.5 p/μL (range 0.2–770) (Fig 2). However, despite the large difference in the range, there was no statistically significant difference between the parasite densities of HTP-LAMP positive and HTP-LAMP negative samples (p = 0.088). HTP-LAMP detected all six samples that had densities greater than 7 p/μL. Among samples with a parasite density of >2 p/μL, HTP-LAMP had a sensitivity of 54% (95% CI 25–81) versus PCR. Among the PCR positive/HTP-LAMP negative samples, 21% (6/29) had parasite densities > 2 p/μL (Table 2). Among samples with a parasite density of ≤ 2 the sensitivity of HTP-LAMP was 36% (CI95% 21–54); 13/36 samples with parasite densities ≤ 2 p/μL were also detected by HTP-LAMP (Tables 1 and 2).

The comparison between HTP-LAMP and Ch-LAMP showed eight samples positive with Ch-LAMP but negative with HTP-LAMP, 5/8 had parasite densities of ≥ 2/μL. Another six were HTP-LAMP positive and Ch-LAMP negative, two of them were also negative by PCR (Tables 1 and 2). The Kappa- agreement between the two LAMP assays was 0.53 (p<0.001).

### Field performance of the HTP-LAMP assay

The HTP-LAMP was performed by two pre-trained lab technicians under supervision of a third person from the research team. The high-throughput sample preparation was experienced as user friendly, with less steps where errors could be introduced than with the boil and spin extraction method. Use of an 8-channel pipette for transferring the DNA eluates to LAMP reaction tubes was beneficial for reducing pipetting errors, compared to the boil and spin method where each sample was pipetted individually. All 96 samples in the 12 strips were read simultaneously under a UV lamp.

The most time consuming part of the work for the two technicians was to cut the samples into the deep-well plates. It took around one hour to sort and cut the 94 sampling devices for one plate. During the following incubation times the technicians could prepare for the next plates. It took three hours from start to results for one plate; three plates were completed in six hours. Two technicians analysed up to 282 samples (3x94 samples) per day.

## Discussion

A newly developed high through-put DNA extraction method for LAMP was evaluated under field conditions in Zanzibar. The PCR and HTP-LAMP determined malaria prevalence among asymptomatic individuals was 1.6% (95%CI 1.3–2.4) and 0.7% (95%CI 0.4–1.1), respectively. The HTP-LAMP evaluated in this study had a sensitivity of 40.8% versus PCR. The sensitivity of HTP-LAMP for detecting low-density infections in this field setting was thus lower than anticipated [12].

The evaluation of molecular methods becomes extremely taxing when approaching the limits of detection. Low parasite densities, with a mean of < 2 p/μL, may be the main explanation for the low sensitivity of HTP-LAMP; the parasite densities were simply below the detection limit of LAMP. However, several parasitemias with densities > 2 p/μL were also missed by both HTP-LAMP and Ch-LAMP. Hence, the sensitivity of HTP-LAMP versus PCR in asymptomatic samples with parasite densities > 2 p/μL was still low (54%) compared to what was found in the evaluation of clinical samples (100%) [12]. On the other hand, both HTP- and Ch-LAMP detected samples with densities as low as 0.2 p/μL. The just slightly higher sensitivity of Ch-LAMP (49%) compared to HTP-LAMP (41%) and a kappa agreement of 0.53 between the two extraction methods suggests that the HTP extraction may not be a major cause of the low sensitivity of HTP-LAMP experienced in this field setting.

Low reproducibility of molecular methods in low density infections may also partly explain the low sensitivity. As shown in this study, parasite densities on the border of the detection limit can alternate between being positive and negative and the reproducibility of results may be low even when repeated from the same blood spot [23,24]. During the evaluation it would have been useful to freeze the remaining HTP eluate to enable PCR and LAMP repeats from the same DNA-extraction. However, the stability of the HTP DNA-extract remains to be investigated. Adding more concentrated DNA or using a larger volume of blood may be options to improve the sensitivity of HTP-LAMP. It may also be recommended for future use to include positive and negative controls in each round of extraction of 96 samples.

In the accompanying article by Perera et al the sensitivity of HTP-LAMP was 78% (95% CI 54–100) for parasite densities < 2 p/μL (geometric mean 1.0 p/μL, range undetected-1.5), and 100% for parasite densities > 2 p/μL (geometric mean 712 p/μL, range 2–912,896), compared to nested PCR. These evaluations were based on clinical samples collected on the same filter paper devices as in the present study but the results were read with a turbidimeter [12]. Previous publications using various LAMP methods, including evaluations of the Loopamp kit, have shown sensitivities of LAMP of > 90% [6,7,10,16,25,26]. The sensitivity of Loopamp for detection of *P*. *falciparum* used together with Chelex-extracted DNA for detection of asymptomatic carriers was 97% (geometric mean parasite density of 10 p/μL, range 0–4972) when compared to PCR [16] and the sensitivity of Loopamp for detection of asymptomatic *P*. *falciparum* infections with boil and spin extraction under field conditions has been 100% (range 1–897 p/μL) and 83% (geometric mean 26 p/μL, range 0–4626) [10,26].

The comparably lower detection limit of PCR represents an advantage for detection of parasites in malaria pre-elimination settings; however, PCR does not yet provide the field applicability and short time to result necessary for antimalarial treatment that LAMP can offer. The HTP-LAMP assay was experienced as easy to set up and to use by the technicians. The sampling devices were also user-friendly offering easy blood sampling and cutting of samples into the wells of the lysis plates. Although the time to result is comparable to the boil and spin method when used in field settings [10,11] the HTP set up was experienced as easier to use with reduced risks of pipetting errors. Up to three plates of 96 samples were analysed per day, although, if available more samples could have been processed in a day.

The use of a UV-lamp for reading results is comparably less objective than a turbidimeter. The difference between a positive and negative result was somewhat subtle with the UV-LAMP set up in almost day light conditions. It is therefore important to use a dark space and a good quality UV-lamp when recording results. The cost of the HTP sample preparation may be estimated to 3 USD per sample which is considerably more expensive than the boil and spin method. The consumables were packed in cardboard boxes, one for each set of 96 samples, constituting a large volume to be shipped by air. Shrinking the size of the packaging of consumables may thus be recommended to enable easier and cheaper transport to field areas.

## Conclusion

Although field applicable, this high throughput format of LAMP as used here was not sensitive enough to be recommended for detection of asymptomatic low-density infections in areas like Zanzibar, approaching malaria elimination.
